# Preventing falls among older people with mental health problems: a systematic review

**DOI:** 10.1186/1472-6955-13-4

**Published:** 2014-02-19

**Authors:** Frances Bunn, Angela Dickinson, Charles Simpson, Venkat Narayanan, Deborah Humphrey, Caroline Griffiths, Wendy Martin, Christina Victor

**Affiliations:** 1 Centre for Research in Primary and Community Care, University of Hertfordshire, Hatfield, Hertfordshire AL10 9AB, UK; 2 Oxford Health NHS Foundation Trust, Fulbrooke Centre, Churchill Hospital, Oxford OX3 7JX, UK; 3 School of Health Sciences and Social Care, Brunel University, Uxbridge UB8 3PH, UK

**Keywords:** Systematic review, Falls, Older people, Mental health

## Abstract

**Background:**

Falls are a leading cause of mortality and morbidity in older people and the risk of falling is exacerbated by mental health conditions. Existing reviews have focused on people with dementia and cognitive impairment, but not those with other mental health conditions or in mental health settings. The objective of this review is to evaluate the effectiveness of fall prevention interventions for older people with mental health problems being cared for across all settings.

**Methods:**

A systematic review of fall prevention interventions for older people with mental health conditions. We undertook electronic database and lateral searches to identify studies reporting data on falls or fall related injuries. Searches were initially conducted in February 2011 and updated in November 2012 and October 2013; no date restrictions were applied. Studies were assessed for risk of bias. Due to heterogeneity results were not pooled but are reported narratively.

**Results:**

Seventeen RCTs and four uncontrolled studies met the inclusion criteria; 11 involved single interventions and ten multifactorial. Evidence relating to fall reduction was inconsistent. Eight of 14 studies found a reduction in fallers (statistically significant in five), and nine of 14 reported a significant reduction in rate or number of falls. Four studies found a non-significant increase in falls. Multifactorial, multi-disciplinary interventions and those involving exercise, medication review and increasing staff awareness appear to reduce the risk of falls but evidence is mixed and study quality varied. Changes to the environment such as increased supervision or sensory stimulation to reduce agitation may be promising for people with dementia but further evaluation is needed. Most of the studies were undertaken in nursing and residential homes, and none in mental health hospital settings.

**Conclusions:**

There is a dearth of falls research in mental health settings or which focus on patients with mental health problems despite the high number of falls experienced by this population group. This review highlights the lack of robust evidence to support practitioners to implement practices that prevent people with mental health problems from falling.

## Background

Falls are the most commonly reported patient-safety incident in mental health settings for older people [1] with approximately 36,000 falls reported in these settings annually in England alone [2]. Similar numbers of falls and injuries in mental health settings are also reported in other countries e.g. Australia [3] and the United States [4]. Risk of falling is exacerbated by mental health problems, such as impaired mental status due to dementia [5], depression [6] mania and anxiety [7]. In addition, treatments of mental health conditions, for example, with psychotropic medication [8,9] and electroconvulsive therapy [10] also increase fall risk. Falls affect rehabilitation, physical and mental function, can increase length of stay in hospital settings and the likelihood of discharge to long-term care settings [11]. Health care costs associated with falls are increasing worldwide [12], with falls in older people with mental health conditions associated with greater costs compared to the general population of older people [4].

A previous systematic review [13] investigated the effects of cognitive impairment, in particular dementia, on strategies to prevent falls and fractures in hospitals and care homes, and included studies that had been undertaken in a range of settings including Accident and Emergency, care homes, acute general hospitals, sub-acute units and rehabilitation wards. However, no studies were reported from mental health settings, and the authors did not explore mental health conditions other than dementia and cognitive impairment. A more recent review [14] also focused on Alzheimer’s disease and related dementias. Although other reviews have looked at fall prevention in hosptial settings [15,16] we did not find any systematic reviews which explored fall prevention interventions specifically in mental health settings or which focused on people with mental health problems. The evidence-base for fall prevention interventions for older people with mental health problems appears to be poorly developed [17,18]. A lack of clear evidence leaves practitioners, particularly nurses who provide the day-to-day care of patients, to struggle to prevent and manage falls in these populations [19].

This paper presents the results of a systematic review where we addressed the following aim: What interventions are effective in preventing and managing falls among older people with mental health conditions.

## Methods

The systematic review was carried out using methodology employed by the review team in previous reviews, and in accordance with that recommended by the Cochrane Collaboration [20]. The inclusion criteria and methods for the review were pre-specified in a protocol (available on request from authors).

### Search strategy and selection criteria

We searched for published and unpublished English language studies that evaluated an intervention aimed at preventing or reducing falls in older people with a mental health problem, regardless of setting. Studies were identified from searching a range of electronic databases including Pubmed, NHS evidence, Cochrane Library inc, Cochrane Database of Systematic Reviews, Database of Abstracts of Reviews of Effects, CENTRAL, NHS Economic Evaluation Database and the Health Technology Assessment Database, Cinahl, AMED, BNI, Embase, HMIC, PsychInfo; and by using lateral search techniques such as checking reference lists, and using the ‘Cited by’ option on Web Of Science (WoS) and Google Scholar, and the ‘Related articles’ option on PubMed and WoS. Searches were first conducted in February 2011 and updated in November 2012 with lateral searching ongoing until October 2013; no date restrictions were applied. Further details of the search terms used can be seen in Table 1.

**Table 1:** Example of search terms

**Database & date searched**	**Search terms**
PubMed (February 2011)	fall*[ti] AND (falls OR accidental falls OR falls in the elderly) AND (mental disorders OR mental health OR dementia OR cognitive impairment OR cognitive disorder OR depression OR paranoia OR personality disorder OR anxiety OR delerium OR amnesia OR parkinsons)

*= Truncatated.

We included both randomised controlled trials and controlled evaluations of fall prevention interventions for people aged 60 and over with any mental health problem including dementia, depression or psychosis. Studies that did not have a specific mental health focus were included as long as a significant percentage (e.g. 50% or more) of participants had a mental health problem, or data was reported separately for those with a mental health condition. As preliminary searches suggested there was a lack of controlled studies we included uncontrolled studies, but only if all participants had a mental health problem. We included single focus or multi-factorial interventions involving environmental, exercise, technological, psychological, educational, and health related components. Our primary interest was in studies delivered in in-patient mental health settings but interventions delivered in other settings were included as long as the study included older people with a mental health problem. The primary outcomes of interest were the number of participants sustaining at least one fall (fallers) and data relating to the rate or number of falls. Secondary outcomes included fall related injuries, hip fractures, service use and patient satisfaction. In addition, we searched for qualitative studies or process evaluations that identified barriers and facilitators to the implementation and uptake of interventions; in particular looking at whether specific guidance is required for this group.

### Data extraction and critical appraisal

Two reviewers independently screened titles and abstracts of citations identified by electronic searches, applied the selection criteria to potentially relevant papers and extracted data from included studies using a standardised form which was piloted prior to use. Any disagreements were resolved by consensus or by discussion with a third reviewer. We extracted information on the type of study design, study aims, participants, setting, intervention (including details of provider, duration and intensity) and outcomes.

Two reviewers independently assessed the quality of included studies. RCTs and controlled studies were assessed using the Cochrane Collaboration risk of bias tool [20], and uncontrolled studies were assessed on domains adapted from two check lists [21,22]. Full details of the quality domains can be seen in Table 2.

**Table 2:** Quality assessment criteria by study type

**Randomised controlled trials all scored as *Yes (+)/No (-)/Unclear***	
*Sequence generation*	Was the allocation sequence adequately generated?
*Allocation concealment*	Was allocation adequately concealed?
*Blinding*	Was knowledge of the allocation intervention adequately concealed from outcome assessors?
*Incomplete outcome data*-	Was this adequately addressed for each outcome?
*Selective outcome reporting*	Are reports of the study free of suggestion of selective outcome reporting?
*Other source of bias*	Was the study apparently free of other problems that could put it at a high risk of bias?
**Uncontrolled before after studies all scored as Yes (+)/No (-)/Unclear**	
*Selection bias*	Are the individuals selected to participate in the study likely to be representative of the target population?
*Blinding*	Was knowledge of the allocation intervention adequately concealed from outcome assessors?
*Detection bias*	Outcomes reported and measured in standardised way
*Incomplete outcome data*	Was this adequately addressed for each outcome?
*Selective outcome reporting*	Are reports of the study free of suggestion of selective outcome reporting?
*Other source of bias*	Was the study apparently free of other problems that could put it at a high risk of bias?

### Analysis

The interventions in the included studies were classified using the fall prevention classification system developed by the Prevention of Falls Network Europe (ProFANE) [23]. This groups studies by setting (nursing/residential care, hospital, community), by combination (single, multifactorial) and by type of intervention including exercise, social environment, environment/assistive technology, knowledge, and other (multi-sensory stimulation). Populations were not homogenous and the Chi-Square test and I^2^ test [24] revealed significant heterogeneity and thus studies were not pooled in a meta-analysis. Instead we have presented data in the text and in a table with an indication of whether the effect of the intervention was positive, negative or not statistically significant. Where possible we have reported dichotomous outcomes as relative risks or incidence rate ratios and continuous data as mean differences, both with 95% confidence intervals. Where data were not available to allow us to calculate effect sizes we have presented data as reported in the paper (for example P values).

## Results

### Selected studies

**Figure 1:**
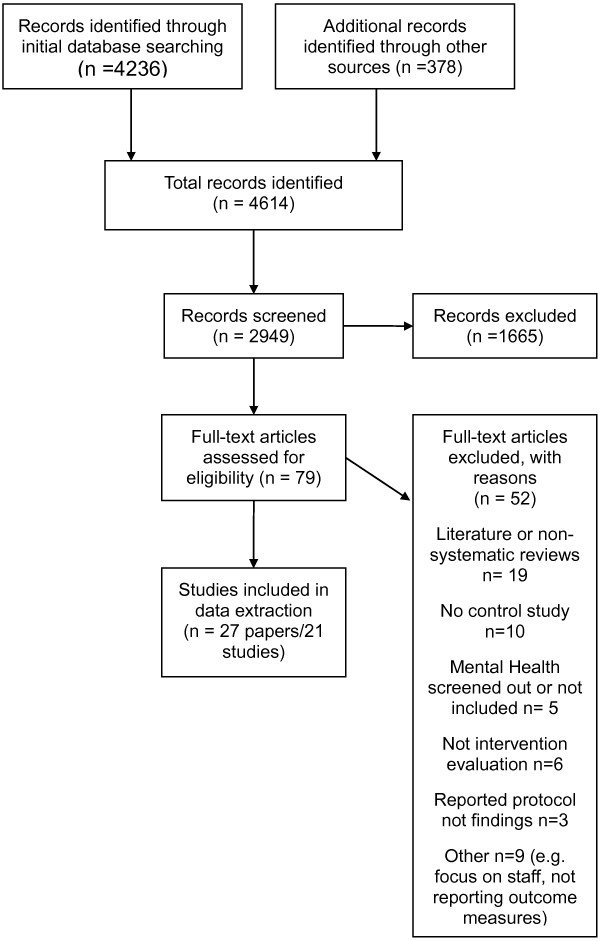
Flow chart of study selection process.

We identified 4614 studies from our electronic and lateral searches of which 27 papers [25-51] reporting 21 studies met the inclusion criteria. Studies were published between 1997 and 2013, with all but one published in the last ten years. Seventeen studies were randomised, with eight being cluster RCTs [26,27,33,34,39,41-43], and nine with randomisation at the level of the individual [28,32,36,38,44,45,48,50,51]. Four studies were uncontrolled [29,30,37,46]. Length of follow up ranged from 3 to 12 months. We found no qualitative studies or process evaluations. A flow chart detailing the identification of studies can be seen in Figure 1.

### Setting and populations

Nine studies were conducted in Europe, five in Australia, five in North America and two in Japan. Fourteen studies took place in nursing homes or residential care [26-30,34,36,39,41-43,45,46], or included participants who mostly resided in nursing or residential care [52], three took place in hospital [33,38,48], one in a respite day centre [37], one a geriatric outpatient clinic [32] and two in participants’ own homes [44,51]. In ten studies participants all (or most) had dementia or cognitive impairment [27-30,36-39,45,51,52], four studies reported sub-group analyses for participants with cognitive impairment [33,35,41] or depression [44] and the rest included 48% or over of people with mental health problems (largely dementia).

### Intervention characteristics

Ten studies involved multi-factorial interventions [32,34,37,39,41,42,44,47,48,51]; and included components such as staff training, physical activity or training and environmental assessment. Of the single component interventions two focused on knowledge based interventions [26,33], five on the environment [27,29,30,38,46] two on physical activity or exercise [28,43], and two on sensory stimulation, one involving , olfactory stimulation with lavender oil [45], and the other multisensory stimulation through a Snoezelen room [36]. ProFANE classifications can be seen in Table 3 and further details of included studies (including the links between papers) in Table 4.

**Table 3:** Overview of setting and intervention type using PROFANE domains

**Setting/*combination***	**Study ID**	**Exercise**	**Medications**	**Management of urinary incontinence**	**Fluid/nutrition therapy**	**Environment/assistive technology**	**Social environment**	**Knowledge**	**Other**
**Nursing/residential care facility**									
***Single***	Bouwen 2008 [26]							****	
	Buettner 2002 [28]	****							
	Chenowith 2009 [27]						****		
	Detweiler 2005 [29]						****		
	Detweiler 2009 [30]					****			
	Klages 2011 [36]								****Multisensory stimulation
	Rosendahl 2008 [43]	****							
	Sakamoto 2012 [44]								****Lavender patches
	Shimada 2009 [45]						****		
***Multifactorial***									
	Jenson 2003 [35]	****	****			****	****		
	Neyens 2009 [40]	****	****			****	****		
	Rapp 2008 [41]	****						****	
	Ray 1997 [42]		****			****	****	****	
	Shaw 2003 [46]	****	****			****			
**Hospital**									
***Single***	Haines 2011 [33]							****	
	Mador 2004 [38]						****		
***Multifactorial***	Stenvall 2007 [47]		****	****	****	****	****		
**Community based**									
***Multifactorial***	Faes 2011 [32]	****						****	
	Mackintosh 2005 [37]	****				****			
	Wesson 2013 [51]	****						****	
	Salminen 2009 [43]	****	****			****		****	

****Intervention type.

**Table 4:** Summary of included studies characterised using PROFANE domains

	**Authors and study design**	**Research question**	**Description of intervention**	**Participants**	**Setting**
**Single interventions**					
***Exercise***					
	Buettner 2002 [28]	Does a therapeutic recreation intervention reduce falls in older adults with dementia?	I = 3 month therapeutic recreation program delivered at time of day and location where falls occurred; to increase strength, endurance, flexibility and balance.	25 people with dementia & history of previous falls. Aged 60+ (mean age 83), MMSE <= 23 (M=2.63)	Nursing Home, America
	RCT (2 months FU)		C = usual activities		
	Rosendahl *et al.* 2008 [43]	Does an exercise program reduce falls in residential care facilities?	I= 3 month individualised weight-bearing exercise intervention	191 people aged 65+ (mean age 85), MMSE 10+ (M=17.8), 52% with dementia	Residential Care, Sweden
	Cluster RCT (6 months FU)		C = non-exercise control activity while sitting		
***Environment/assistive technology***					
	Detweiler *et al.* 2009 [30] (also Detweiler 2008) [31]	Does a dementia wander garden and medication review reduce number and severity of falls?	I = wander garden and medication review	28 people with dementia aged 74-92 (mean age 81).	Residential Dementia Care unit, America
	Uncontrolled before/after study (12 months FU)				
***Social environment***					
	Detweiler *et al.* 2005 [29]	Does consistent supervision during day and evening shifts reduce falls in dementipea unit?	I = Supervision focusing on behavioural and environmental factors.	8 older people with dementia aged 74 to 85 (mean age 81)	Dementia Care Home, America
	Uncontrolled before/after study (4 month FU)				
	Shimada *et al.* 2009 [45]	Does a falls prevention aide using systematic supervision reduce falls?	I = Aide delivered intervention, targeting residents considered to be at high risk of falls	60 people aged 68-105 (mean age 87), 48% Dementia, 5% cognitive impairment , 2% depression	Long-term aged-care facility, Japan.
	Uncontrolled before/after study (25 week FU)				
	Chenowith *et al.* 2009 [27]	Investigate effectiveness of person-centred care and dementia-care mapping compared with each other and with conventional dementia care	I 1= Person centred care	296 Average age: 83 for dementia care mapping 84 for person centred care 85 usual care	Residential Care sites, Australia
	Cluster RCT (4 Month FU)		I 2= Dementia care mapping.		
			C=Usual care.		
	Mador *et al.* 2004 [38]	Does individualized advice on non-pharmacological strategies for hospitalized older patients with confusion and behavioural problems improve levels of agitation and reduce the use of psychotropic medication.	I= Patient assessment, non-pharmacological management plan, on-going support and education for nursing staff. Tailored to patient needs-included addressing patient safety, minimising restraint use, reducing fall risk, communication, behavioural strategies and education.	71 older people with confusion	Acute Hospital, Australia
	RCT (FU to discharge)		C= usual care- included review with geriatrican.	Mean age I=82, C=83	
***Knowledge***					
	Bouwen *et al.* 2008 [26]	Does a staff-oriented intervention impact on the number of accidental falls in residents with and without cognitive impairment?	I = 6 wk multifaceted intervention involving staff training on falls risk factors, followed by a falls diary and patient questionnaire linking risk with possible interventions.	379 older people with mean age of 83 and MMSE <23 (M=15.72)	Nursing Home, Belgium
	Cluster RCT (6 month FU)		C = no staff training, no diary, no questionnaire		
	Haines *et al.* 2011 [33]	Evaluative comparison of 2 forms of multimedia patient education intervention alongside usual care for the prevention of falls.	I1 = written and video based intervention materials and 1-to-1 follow-up with a physiotherapist, in addition to usual ward based care (median time spent with patient 25 (20-36) minutes, maximum with one patient 200 minutes).	1206 people aged 60 + (mean age 75), mean SPMSQ = 8.4, 25% cognitive impairment	In-patient, Australia
	Cluster RCT (FU to discharge)		I2 = intervention materials provided but without 1-to-1 with physiotherapist, in addition to usual ward based care.		
			C = usual ward based care		
***Other***					
Multisensory stimulation	Klages et al. 2011 [36]	To investigate the influence of multisensory stimulations in a Snoezelen room on the balance of individuals with dementia.	I= 30 mins use of a Snoezelen room twice a week for 6 weeks.	19 older people, mean age 86. MMSE 12 (range 4-22) for IV group, 13 (2-22) for control. Able to walk with minimal assistance and understand simple instructions.	Long term care home, Canada
	RCT (FU 6 weeks post intervention)		C= volunteer spending same amount of time 1-to-1 with resident.		
Multisensory stimulation	Sakamoto *et al.* 2012 [44]	Does a lavender olfactory stimulation intervention reduce falls in nursing home residents?	I = 12 month, 24 hour exposure to lavender olfactory stimulation patch on clothes near neck	145 people aged 65+ (mean age 84), mean MMSE = 15. able to transfer independently	Nursing Home, Japan
	RCT (360 days FU)		C = same patch and duration as intervention, but no lavender		
**B: Combination interventions: multiple**					
	Wesson 2013 [51]	To test design and feasibility of a home hazard reduction and balance and strength exercise fall prevention program for people with mild dementia living in the community.	I= Strength & balance training, home hazard reduction, discussion of behaviour and management issues with carers. Carers supervised exercise and responsible for implementation of home safety recommendations.	11 patient and carer dyads.	Community, Australia
	RCT (4 month FU)		C= Usual care.	Mean age I= 78.7, C=80.9	
			Both groups received health promotion brochures on fall prevention and home safety.		
	Faes *et al.* 2011 [32]	Is a multifactorial fall prevention program more effective than usual geriatric care?	I = Psychological training for staff & physical training for patients	33 older people, mean age 78, mean MMSE 25, 48% had mild cognitive impairment or dementia	Geriatric outpatient clinic, The Netherlands
	RCT (6 month FU)		C= Usual geriatric care		
	Jensen *et al* 2002 [34], Jensen et al. 2003 [35]	Does a multi-factorial intervention reduce falls & fall-related injuries, in a high risk population in residential care?	I = 11 week multifactorial intervention including staff education, & resident exercise	40 people aged 65-100 (mean age 83), mean MMSE = 19, 36% with dementia	Residential Care, Sweden
	Cluster RCT (34 week FU)		C = Usual care, no staff education.		
	Mackintosh *et al.* 2005 [37]	How feasible and effective is a falls-prevention programme for community dwelling people with dementia?	I = Multifactorial including individualised management plan, mobility exercises, foot health, and multidisciplinary referrals.	64 people with dementia aged 53-93 (mean age 80)	Respite Day Centre, Australia
	Uncontrolled before/After study (6 month FU)				
	Neyens *et al.* 2009 [39] (also Neyens 2006 [40])	Is a multidisciplinary fall prevention intervention effective for psychogeriatric nursing home patients?	I = 12 month multifactorial intervention including assessment & evaluation	518 people with dementia, mean age 82	Psychogeriatric nursing homes, The Netherlands
	Cluster RCT (12 months FU)		C= Usual care, staff had no insight in the fall prevention programme.		
	Rapp *et al* 2008 [41] (also Becker *et al.* 2003 [25])	Is a multifactorial fall prevention program effective in pre-specified subgroups of nursing home residents?	I = 12 month intervention including staff training & education and exercise, and environmental assessments for residents	725 people >60 (mean age 86), 46% with cognitive impairment.	Long-term Nursing Homes, Germany
	Cluster RCT (12 months FU)		C= No specific fall prevention measures		
	Ray *et al.* 1997 [42]	Does a safety intervention prevent falls and associated injury in high-risk nursing home residents?	I = Individual and facility-wide safety and environmental assessment	482 people aged >65 (mean age 83), 49% with cognitive impairment	Nursing Home, America
	Cluster RCT (12 months FU)		C = no assessments or activities		
	Shaw *et al.* 2003 [46]	Does a multifactorial intervention reduce falls in older patients with cognitive impairment and dementia attending an accident and emergency department?	I = Multidisciplinary risk assessment and intervention	274 people aged 65+ with MMSE <24, 89% with dementia.	Community, UK
	RCT (12 months FU)		C = Assessment but no intervention		
	Stenvall *et al.* 2007 [47]	Does a post-operative multidisciplinary, multifactorial intervention reduce inpatient falls and fall-related injuries in patients with femoral neck fracture?	I = Post-op care in geriatric ward with special intervention programme (staff education, joined up assessments by OT and dietician)	199 older people aged 70+ (mean 82), 33% with dementia and 33% with depression.	Orthopaedic and geriatric hospital departments, Sweden.
	RCT (follow up not clear)		C = Conventional care in orthopaedic ward		
	Salminen *et al.* 2009 [43]	Does a multi-factorial fall prevention program reduce falls and which subgroups benefit the most?	I = 12 month intervention based on individual patient risk analysis.	591 people aged 65+, with at least one fall in previous year, and able to walk 10 metres	Community, Finland
	RCT (12 months FU)		C = initial counselling and guidance but no follow up over the 12 month period	52 people in I with GDS ≥11, 40 in control group.	

*I*, intervention; *C*, control; *M*, mean; *MMSE*, mini mental state examination.

### Outcomes

All 21 included studies reported falls data in some form, although seven did not include a specific definition of a fall [27,28,30,36-38,46]. In the others a fall was defined as unintentionally [34,41-44,48,51], inadvertently [33,39,45,47], or unexpectedly [32] coming to rest on the ground, floor, or a lower level; whether or not an injury was sustained [34,43,44,47]. One study included syncopal falls [48], one both witnessed and non-witnessed falls [29]; one only took account of falls where medical intervention was needed [26], and one categorised injurious falls as those resulting in serious injuries that received medical treatment [42].

### Risk of bias

**Figure 2:**
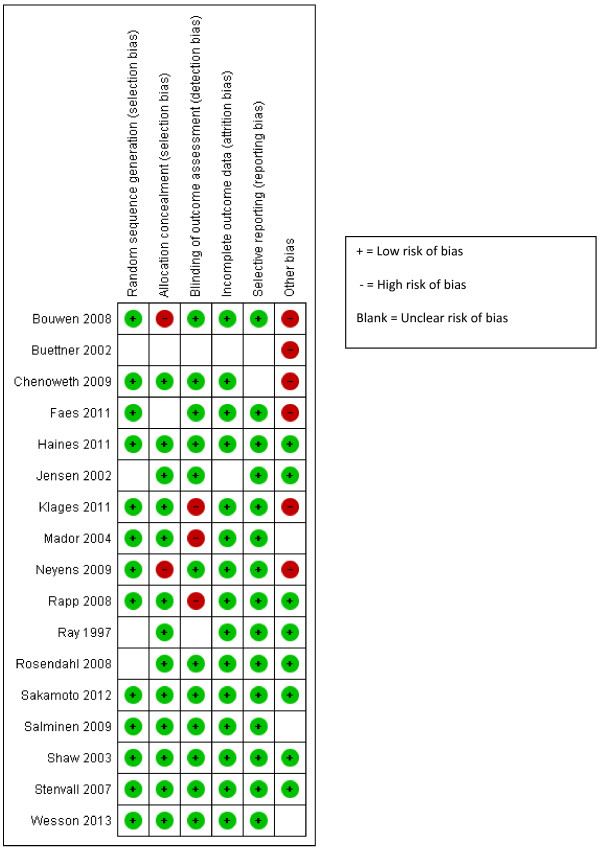
Risk of bias summary: review authors’ judgements about risk of bias item for each included study (RCTs).

**Figure 3:**
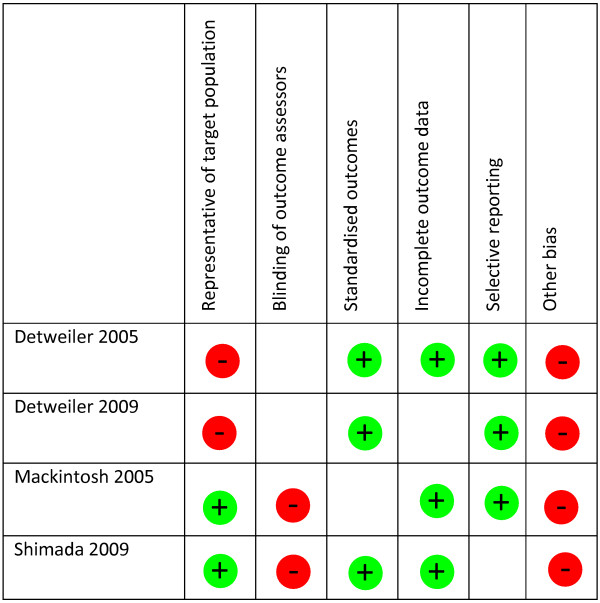
Risk of bias summary: review authors’ judgements about risk of bias item for each included study (Uncontrolled studies).

Results of the risk of bias assessment can be seen in Figure 2 (RCTs) and Figure 3 (uncontrolled studies). Of the 17 randomised controlled studies sequence generation was considered adequate in thirteen studies, as was allocation concealment. However, only ten studies were judged to have both adequate sequence generation and allocation concealment [27,33,36,38,41,44,45,47,48,51]. Four studies [33,45,47,48] met all quality criteria and six met five or more of the six quality criteria [32,41,43,44,47,51]. One RCT [28] met none of the criteria. Of the four uncontrolled studies three met three out of six of the criteria [29,37,46] and one met two out of six [30], but all are at high risk of bias because they have no control group.

### Evidence of effectiveness

Results are presented by setting and according to whether the intervention was single or multifactorial. A summary of the data, including effect sizes, can be seen in Table 5. Study quality is reported in the text for those studies judged to be at low or high risk of bias; all quality scores can be seen in Table 5.

**Table 5:** Overview of main results

**Intervention/study**	**Fallers (fell at least once)**	**Rate/number of falls**	**Fall related injuries/fractures**	**Other**	**Quality score**
**Nursing/residential care: single**					
**Exercise**					
Buettner 2002 [28]^2^	Reduced*; RR 0.57 95% CI 0.37, 0.89				0/6
Rosendahl 2008 [43]^1^	*No difference;* RR 1.04 95% CI 0.78, 1.37	*Reduced*; IRR 0.82 (0.49-1.39)	Hip fractures –*Reduced* RR 0.16 95% CI 0.01, 3.01		5/6
**Social environment**					
Detweiler 2005 [29]^2^	Falls (total number): Reduced* P = 0.024			Fall severity – *No difference*	3/6^4^
Chenoweth 2009 [27]^2^		DCM v control Reduced* p = 0.02			4/6
		PCC v control Increased* p = 0.03			
Shimada 2009 (45)^1^	Reduced* P = 0.012	***Reduced*** * p = 0.046			3/6^4^
**Environment/assistive technology**					
Detweiler 2009 [30]^2^		Reduced* P = 0.05			2/6^4^
**Knowledge**					
Bouwen 2008 [26]^1^	Reduced*; RR 0.57 (0.37, 0.89)	*No difference* p = 0.10			4/6
**Sensory**					
Klages 2011 [36]^2^		*No difference*; p = 0.47			4/6
Sakamoto 2012 [44]^1^	*Reduced:* RR 0.71 95% CI 0.48,1.05	***Reduced*** * IRR 0.57 (95% CI 0.32-0.99)			6/6
**Nursing/residential care: multifactorial**					
Jensen 2003 [35]^3^	CI: *No difference p = .352*		Injuries		
			CI grp – *No difference* IRR 0.9 (95% CI 0.5-1.3)		
	No CI: *Reduce*d* p = .020				
			No CI grp – *No difference* IRR 0.9 (95% CI 0.5-1.5)		
			Hip fractures		
			CI group Reduced* p = .006		
Neyens 2006 [40]^2^		***Reduced*** *; RR 0.64 95% CI 0.43, 0.96			4/6
Rapp 2008 [41]^3^; Becker 2003^1^		Cognitively impaired^3^	Hip fractures^1^ – *Increased*	Time to first fall^3^ (cognitively impaired versus cognitively intact) – Increased*	5/6
		***Reduced*** *; Incidence rate ratio (IRR) 0.43 (0.28-0.66)	RR 1.11 95% CI 0.49, 2.51		
		Depression (at least one sign)		RR 0.49 95% CI 0.35, 0.69	
		*Reduced;* IRR 0.74 (0.51-1.09)			
Ray 1997 [42]^1^			Injurious falls	Recurrent fallers – reduced	4/6
			Reduced		
			RR 0.75 95% CI 0.48, 1.17	RR 0.83 95% CI 0.68, 1.02	
Shaw 2003 [46]^2^	*Reduced*; RR 0.92 95% CI 0.81, 1.05			Fall related A&E admissions – increased	6/6
				RR 1.25 95% CI 0.91, 1.72	
**Hospital: single**					
**Knowledge**					
Haines 2011 [33]^3^	Complete intervention vs control	Complete intervention vs control	Injuries	Cognitively impaired	6/6
			Increased	Proportion who fell *no difference* – complete program 26%, control 24%	
			RR 1.84 95% Cl 0.93, 3.62		
	*Increased*; 1.38 (0.70, 2.75)	*Increased;* HR 1.48 (0.86-2.53)			
	Materials only vs control	Materials only vs control			
	*Decreased*; OR 0.92 (0.48-1.78)	*No difference;* HR 0.99 (0.55-1.78)			
**Environment (social)**					
Mador 2004 [38]^2^	Increased; RR 2.43 (0.84-7.03)				4/6
**Hospital: multifactorial**					
Stenvall 2007 [47]^1^	Reduced*; RR 0.78 (95% CI 0.64, 0.96)	Reduced* IRR 0.38 (95% CI 0.20-0.76)^1^	Injuries & serious injuries		6/6
		Reduced* IRR 0.07 (95% CI 0.01-0.57) - PWD	Reduced* (p = 0.002, p = 0.055)		
**Community based: multifactorial**					
Faes 2011 [32]^1^	*Increased* RR 1.39 95% CI 0.66, 2.93	*Increased* RR 2.12 (95% CI 0.6 – 7.56) p = 0.25			4/6
Mackintosh 2005 [37]^2^	*No difference* p = 0.27				3/6^4^
Wesson 2013 [51]^2^	*Reduced*; RR 0.50 (95% CI 0.11, 2.19)	*Reduced*: IRR 0.34 (0.06, 1.91)			5/6
Salminen 2009 [43]^3^		***Reduced*** * in participants with higher N depressive symptoms			5/6
		IRR 0.50 (0.28-0.88)			

*statistically significant.

*DCM*, dementia care mapping; *PCC*, person centred care; *CI*, cognitive impairment; *PWD*, people with dementia.

^1^data presented for whole population (e.g. both cognitively impaired and cognitively intact).

^2^Participants all (or most) have mental health problems.

^3^data presented for subgroup with mental health problem such as cognitive impairment or depression.

^4^Uncontrolled study.

### Nursing/residential care: single interventions

Two studies looked at exercise programmes in nursing or residential care. One [28] (high risk of bias) found a significant reduction in numbers of fallers and the other (low risk of bias) [43] no difference in number of fallers but a non-significant reduction in rate of falls and hip fractures. Four studies looked at some form of environmental intervention. One, a study of a wander garden found a significant reduction in the mean number of falls [30] but this was an uncontrolled study and at high risk of bias. Of the other social environmental interventions two studies involving increased supervision [29,46] reported a significant reduction in number of fallers, but both were at high risk of bias. A study comparing dementia care mapping (DCM), person-centred care (PCC) and usual care found a reduction in falls in the DCM group compared to control but an increase in the PCC group compared to control [27]. One study which involved a knowledge based intervention for staff reported a significant reduction in fallers but no difference in the rate of falls [26]. Two studies evaluated a sensory intervention. One study of lavender patches (low risk of bias) found a non-significant reduction in number of fallers and a significant reduction in incidence of falls [45] and a study of multisensory stimulation in a Snoezelen room found no difference in the number of falls in the intervention group compared to control [36].

### Nursing/residential care: multifactorial interventions

Five studies looked at multifactorial interventions in nursing homes or residential care. Common components of these interventions were exercise, medication management, changes to the environment and activities to improve staff knowledge. Two studies report a significant reduction in the rate of falls [40,41] in participants with cognitive impairment. However, in the latter there was a non-significant increase in the number of hip fractures in the intervention group compared to control. One low risk of bias study found a slight (non-significant) reduction in fallers but a non-significant increase in fall related A&E admissions [52], and one a non-significant reduction in injurious falls and number of recurrent fallers [42]. One study which undertook subgroup analyses for participants with cognitive impairment found that the intervention significantly reduced the number of fallers in those with no cognitive impairment but not in the group with cognitive impairment [35].

### Hospital: single interventions

Neither of the hospital based studies found a reduction in falls in those with cognitive impairment. A study of multimedia patient education (low risk of bias) compared multi-media education with health professional follow-up, multi-media materials alone and usual care [33]. Although the complete intervention reduced falls amongst participants who were cognitively intact this was not the case for those with cognitive impairment. Amongst those with cognitive impairment there was an increase in the number of fallers, fall incidence and injurious falls in the group that received the complete intervention but no difference in fallers or fall rate in the group that received materials only. The other hospital based RCT evaluated the impact of nurse led individualised advice to staff aiming to reduce agitation and the use of psychotropic medication in patients with confusion and behavioural problems. They found a non-significant increase in falls in the intervention group compared to control [38].

### Hospital: multifactorial interventions

There was one (low risk of bias) multifactorial hospital based intervention [48]. They found a significant reduction in fallers and in fall incidence in people with dementia. There was also a reduction in injuries and serious injuries.

### Community: multifactorial interventions

Four studies took place in the community. Only one [44], a multifactorial fall prevention program, found a significant reduction in falls. Although the study did not focus on people with a mental health problem they do report results of a sub-group analysis in which they found a reduction in the incidence of falls in people with depressive symptoms. A pilot RCT found a non-significant reduction in fallers and incidence of falls in older people receiving a home hazard reduction and exercise fall prevention programme [51], an uncontrolled pilot study of a fall prevention programme for people with dementia found no difference in fallers [37] and a multifactorial intervention in a geriatric outpatient clinic reported a non-significant increase in fallers and falls [32].

## Discussion

### Summary of main results

We found 17 RCTs and four uncontrolled studies that evaluated the impact of interventions to prevent falls in older people with mental health problems, including dementia, delirium and depression. The nature of the interventions varied considerably and involved a variety of components including physical activity and exercise, risk assessment, environmental modification, staff training, increased supervision, patient education, and sensory interventions. Evidence relating to fall reduction was inconsistent. Of the 14 studies that reported the number of fallers, eight found a reduction which was statistically significant in five, and of the 14 that reported rate or number of falls eight found some evidence of a significant reduction. However, four studies found a non-significant increase in falls in the intervention group compared to the control.

Although all the studies included participants with mental health problems only ten studies had a specific mental health focus. Studies predominantly focused on cognitive impairment, dementia and depression and there were no studies including patients with other psychiatric disorders who are likely to be on medications associated with increased risk of falls. The majority of studies were undertaken in nursing and residential homes, and there were none in mental health inpatient settings.

### Comparison with other literature

Cochrane reviews looking at fall prevention interventions for older people have found evidence that multifactorial interventions are effective in preventing falls in community dwelling participants and also those in hospitals and nursing care facilities, and that multi-component exercise is effective in community settings [53,54]. We also found evidence to suggest that multifactorial interventions which included exercise can be effective in older people with cognitive impairment and depression, with four of six studies finding a reduction in falls which was significant in two [40,48] although there is insufficient evidence to support physical activity or exercise alone with this population.

Interventions that involved changing the social environment through increasing staff knowledge and awareness, levels of supervision and reducing agitation in patients with dementia through improving psychosocial care or using aromatherapy looked promising for this patient group. However most of these studies are uncontrolled and these interventions require further evaluation. Moreover, studies focusing on changes to the social environment present numerous difficulties for implementation as they are reliant on the motivation of *individual* staff to deliver recommended changes to care. In addition, there are problems with measuring variation in care practices over time and adherence to the intervention. Future studies of this type should include a health economic evaluation, as increased supervision will require additional staff.

Only six of the studies, all multifactorial in design, included medication review. This is surprising given that the American Geriatric Society and British Geriatric Society [55] in their Clinical Practice Guideline for fall prevention, argue that the strongest risk associations for falls are with psychotropic medication and polypharmacy and that evidence supports withdrawal or reduction of psychotropic medication.

Although the evidence from this review is not robust it does challenge the American Geriatrics Society and British Geriatrics Society guideline [55] which states that there is insufficient evidence to support *any* recommendation to reduce fall risk for older people with cognitive impairment. However, the review does support current UK guidance from the National Institute for Health and Care Excellence [56] which recommends that all older people at increased risk of falling are considered for individualised multifactorial interventions including strength and balance training, home hazard assessment, vision assessment and medication review; and that those in in care settings such as care homes receive multifactorial interventions including exercise. In addition, all older people taking psychotropic medication should have this reviewed and where possible discontinued. NICE guidance also recommends that health care professionals dealing with patients at risk of falling should be educated in falls assessment and prevention. A recent audit of UK inpatient NHS settings that included mental health settings [57] found that 35% of nurses and 61% of doctors had not received training in fall prevention in the past year thus highlighting that work is needed in order to meet NICE recommendations.

### Limitations and quality of the evidence

We used systematic and rigorous methods to synthesize the current evidence on the effectiveness of fall prevention interventions for older people with mental health problems. However, there are a number of methodological issues that could have a bearing on the validity of these results. Due to the scarcity of evidence relating to falls prevention in older people with mental health problems we included four uncontrolled studies which, because they are at increased risk of bias, should be interpreted cautiously. Moreover, only four of the controlled studies met all of the methodological criteria on our checklist and many appeared underpowered to detect a reduction in falls. We included seven studies that included participants with and without mental health problems and which did not report sub-group analyses for those with mental health problems. It is possible that the results from these studies are less generalizable to older people with mental health problems.

The review involved a diverse range of interventions, participants and outcomes and in light of this heterogeneity we judged meta-analysis to be inappropriate. This made direct comparisons between studies more difficult. Nevertheless, despite this, we were still able to make judgements about the strength and consistency of the findings.

### Difficulties undertaking research with older people with mental illness

The lack of studies we found could reflect the additional methodological and ethical challenges which work in these settings and with these population groups entails. Researchers have highlighted the difficulties of undertaking studies with older patients with mental health problems [41,52], in particular when there are issues around capacity to give informed consent [58]. However, the UK Mental Capacity Act [59] provides researchers with the means to address this, through using assent, though the process adds time and thus cost to the research. Despite this, researchers need to overcome these difficulties in order to provide practitioners with the evidence of effectiveness they need for practice.

Older people with mental health problems are likely to be older (as incidence of dementia increases with age) and frail with physical and mental health co-morbidities and this needs to be accounted for when designing interventions. The authors of one of the studies included in our review [32] suggest that their intervention failed because it was too complex and specialised and that interventions need to be tailored to the needs of frail older people, particularly when incorporating intensive physical therapy.

### Implications for practice and research

Alderson and Roberts [60] argue that reviews such as this one, where the review uncovers very little evidence which practitioners can use, still need to be reported. They argue that uncovering uncertainty can help to improve the evidence base through stimulating more and better quality research.

Staff working with patients with mental health conditions need access to evidence to support their practice with this population group. Staff may be aware of the risks factors for falls, however strategies to prevent falls frequently include choosing management strategies such as restraint and observation [19,61] rather than interventions which would increase balance and strength. In addition, in-patient mental health settings present unique patient-safety issues [62]. Without evidence of effectiveness of interventions staff will continue to struggle to provide care for those at risk of falls who also have a mental health issue such as dementia [19]. Further work is needed to help us to understand what interventions work in which sub groups of patients. This review therefore adds further weight to the calls for further research to help understand which elements of multifactorial interventions work, in what combination for specific groups of patients, the acceptibility of these to older people, and their cost-effectiveness [41,52,55,56].

## Conclusions

Despite the high number of falls experienced by older people with mental health problems, including dementia, we found very few studies reporting fall reduction interventions in mental health settings. No studies were found in mental health hospital settings. The evidence provided in this review does not provide sufficient robust evidence to produce specific guidance for practitioners providing care for older people with the range of mental health problems or in mental health settings. However, it does challenge guidance that states there is no evidence that fall prevention interventions can be effective in older people with cognitive impairment (American Geriatrics Society, British Geriatrics Society 2010). This review highlights the urgent need for further research to develop a robust evidence base to determine which interventions work, in which settings and for whom. This review suggests that the recommendations of UK NICE, that older people should be offered multifactorial interventions including strength and balance training, home hazard assessment, vision assessment, and medication review are applicable to older people with mental health problems [56]. Measures to improve the psychosocial care of older people with dementia also show potential. Staff working with older people at risk of falls should update their knowledge and skills regarding causes and prevention of falls.

## Competing interests

We have no known conflicts of interests.

## Authors’ contributions

All authors were involved in protocol development, study screening and data extraction. FB, AD, CS & CV analysed the data and FB, AD and CV wrote the paper. All authors critically reviewed the paper. All authors read and approved the final manuscript.

## Pre-publication history

The pre-publication history for this paper can be accessed here:

http://www.biomedcentral.com/1472-6955/13/4/prepub
